# Management challenges in a schizophrenic patient with multiple brain abscesses: A case report

**DOI:** 10.1192/j.eurpsy.2021.2118

**Published:** 2021-08-13

**Authors:** M. Huete Naval, P. Albarracin, R. Galeron, E. Herrero, L. Gallego Deike

**Affiliations:** 1 Psychiatry And Mental Health, Hospital Clínico San Carlos, Madrid, Spain; 2 Psychiatry And Mental Health, Hospital Clinico San Carlos, Madrid, Spain

**Keywords:** brain abscess, drug interaction, rifampicine, schizophrénia

## Abstract

**Introduction:**

Cerebral abscesses are rare, occurring in approximately 0.3–1 per 100 000 patients. Mortality rate still remains as high as 22%. Very few cases of acute psychotic episodes associated with brain abscess have been reported.

**Objectives:**

To present a case report of a patient with schizofrenia associated with multiple brain abscesses, focusing on clinical features and managing challenges.

**Methods:**

Presentation of a clinical case supported by a non-systematic review of literature containing the key-words “brain abscess”, “psychosis” and “schizophrenia”

**Results:**

This is a case report of a male 44-year-old patient with a known history of schizophrenia since the age of 18 and with multiple brain abscesses diagnosed 2 month ago. He was admitted to our inpatient service after discontinuation of her medication resulting in an acute psychotic episode. Antibiotic therapy with rifampicine, metronidazole, trimethoprim and sulfamethoxazole was started. Also, administration of clozapine was initiated (up to 400mg/day) with partial improvement, so aripiprazole was added (up to 45 mg/day), with insufficient response. We suspected of a drug interaction between rifampicine (known potent broad inducer of drug-metabolizing enzymes) and antipsychotic medication, so we decided to change aripiprazole to amisulpride 1200 mg/day, which CYP-catalyzed metabolism appears to be minor. A significant improvement in positive symptoms and mood was observed. The patient has since had no delusions or hallucinations and is living independently at home.

**Conclusions:**

This clinical case highlights the possible association between brain abscesses and relapses in schizophrenia. It is of utmost importance to be aware for possible drug interactions between antibiotic therapy and antipsychotic medication.

**Disclosure:**

No significant relationships.

